# Evaluating the short-term and long-term therapeutic effects of immunoadsorption compared with plasma exchange in chronic inflammatory demyelinating polyneuropathy: a long-term, prospective, observational study

**DOI:** 10.1016/j.eclinm.2025.103742

**Published:** 2026-01-08

**Authors:** Zeynep Elmas, Christine Herrmann, Kathrin Kramer, Oender Soylu, Vanessa Roemer, Luisa Jagodzinski, Lars Richter, Maximilian Wiesenfarth, Tanja Fangerau, Stefanie Jung, Regina Gastl, Angela Rosenbohm, Jochen H. Weishaupt, Makbule Senel, Benjamin Mayer, Hayrettin Tumani, Johannes Dorst

**Affiliations:** a Department of Neurology, University of Ulm, Ulm, Germany; b Institute for Epidemiology and Medical Biometry, University of Ulm, Ulm, Germany

**Keywords:** Chronic inflammatory demyelinating polyneuropathy, CIDP, Immunoadsorption, Long-term effects

## Abstract

**Background:**

Chronic inflammatory demyelinating polyneuropathy (CIDP) is an autoimmune-inflammatory disease of the peripheral nervous system. Corticosteroids, intravenous immunoglobulins (IVIGs), and plasma exchange (PLEX) constitute the main therapeutic options, but immunoadsorption (IA) represents a noteworthy alternative to PLEX due to its excellent tolerability and its capability to remove higher rates of autoimmune antibodies. However, evidence of its therapeutic value in CIDP is low, while the effect of repeated IA as long-term therapy is largely unknown. Therefore, in this study, we sought to evaluate the short- and long-term therapeutic effects of IA in CIDP compared to PLEX and preceding therapies (IVIGs and corticosteroids).

**Methods:**

Between 02/12/2013 and 03/03/2025, we prospectively evaluated the course of disease of patients with CIDP who received at least one cycle of IA or PLEX via Shaldon catheter or arteriovenous shunt. All patients were treated in the Department of Neurology of Ulm University, Germany. All patients fulfilled the diagnostic criteria of typical CIDP according to the European Academy of Neurology/Peripheral Nerve Society (EAN/PNS) guideline on diagnosis and treatment of CIDP, had a continuously progressive course of disease, and had previously received treatment with steroids, IVIGs, or both, with insufficient response. All eligible patients who gave their informed consent were selected for the study. Participant data were collected and retrieved using individual case report forms and medical records. One cycle of IA or PLEX consisted of 5 treatments on 5 consecutive days. As the primary outcome parameter, we used a combined CIDP score of 3 validated scales comprising disability (Inflammatory Neuropathy Cause and Treatment (INCAT) score), motor score (Medical Research Council, MRC), and vibration sensitivity (tuning fork test). For short-term effects, we compared absolute CIDP scores 3 days before and directly after the last treatment of each cycle; for long-term effects, we compared changes of CIDP score per month during IA or PLEX compared to preceding treatments in patients with an observation period of at least 6 continuous months under IA or PLEX. We systematically evaluated adverse events (AEs), and collected safety laboratory data as well as immunoglobulin (Ig) reduction levels.

**Findings:**

In total, 80 patients were included, of which 74 received at least one cycle of IA, 25 received at least one cycle of PLEX, and 19 received at least one cycle of both IA and PLEX and were considered for both treatment groups for respective analyses. 41 IA patients and 16 PLEX patients received 2 or more cycles (median 4 (IQR 2–7.5), maximum 43 cycles) over a median time period of 12.0 (6.0–34.0) months in median time intervals of 2.5 (1.9–4.3) months. We observed improvements of CIDP scores post-treatment in the IA group (median improvement from 310 (224–374) to 321 (234–373) points; median difference 5.0 (95% CI 2.0–6.0), p < 0.0001), but not in the PLEX group (median 254 (214–358) vs. 254 (209–351); median difference 0.0 (95% CI 0.0–5.0, p = 0.12). Long-term progression rates compared to preceding corticosteroid and IVIG therapies decreased from 3.8 (2.2–9.1) to 0.2 (−0.5–2.2) points/month in the IA group (median difference −4.0 (95% CI −6.9 to −1.9), p < 0.0001), and from 4.2 (2.6–17.2) to −1.1 (−1.6–0.5) points/month in the PLEX group (median difference −6.3 (95% CI −19.9 to −1.9), p = 0.0010), corresponding to a clinical stabilization of disease progression. We detected 12 (5.0%) asymptomatic and 3 (1.3%) symptomatic thromboses of the jugular veins associated with Shaldon catheter placement among 240 cycles of IA, and 6 (7,5%) thromboses of the jugular vein (all asymptomatic) among 79 cycles in the PLEX group, representing the main complication for both procedures. In both groups, hypoproteinemia (IA 100%, PLEX 93.5%), thrombocytopenia (IA 33.6%, PLEX 4.9%), and anemia (IA 21.6%, PLEX 61.8%) were the most common laboratory findings during treatment.

**Interpretation:**

Our results suggest that repeated IA and PLEX might constitute promising therapeutic options in patients with CIDP who do not sufficiently respond to corticosteroids and IVIGs, stabilizing the disease in the majority of patients. However, its invasive nature and significant risk of jugular vein thromboses must be considered. Study limitations include non-randomised group allocation, retrospective collection of data related to preceding treatments, the possibility of sub-optimal treatment pre-baseline, the limited number of patients, and potential interfering elements (such as cross-overs between IA and PLEX) caused by the long observation time. Because of these limitations, study results do not allow a direct comparison of effectiveness between IA and PLEX, and further randomised controlled trials are needed in this regard.

**Funding:**

This was an investigator-initiated study without institutional or industrial funding.

**Research in context**

**Evidence before this study**

We searched PubMed for reviews, observational studies, clinical trials, and cohort studies investigating efficacy and/or safety of immunoadsorption in CIDP published up to August 1st, 2025, using the terms “chronic inflammatory demyelinating polyneuropathy” or “CIDP” in combination with “immunoadsorption”. After reviewing the abstracts, we identified 2 case reports, 6 retrospective case series, and 1 randomised controlled trial evaluating short-term effects vs. IVIGs (10 vs. 10). Only one case series reported effects of repeated IA cycles over an observation period of 6 months.

**Added value of this study**

This observational, prospective study investigating efficacy and safety of repeated IA and PLEX cycles in 80 patients over 10 years provides preliminary evidence that long-term IA and PLEX are capable of stabilizing the course of disease in progressive cases with insufficient treatment response to IVIGs and/or corticosteroids, while yielding moderate, but significant beneficial short-term effects after each cycle. While procedure-specific tolerability was excellent, relevant side effects related to the necessary venous access were detected.

**Implications of all the available evidence**

This study suggests that implementation of repeated IA or PLEX cycles may constitute a promising treatment alternative in therapy-refractory patients with CIDP. Study limitations include non-randomised group allocation, retrospective collection of data related to preceding treatments, the possibility of sub-optimal treatment pre-baseline, the limited number of patients, and potential interfering elements (such as cross-overs between IA and PLEX) caused by the long observation time. Because of these limitations, study results do not allow a direct comparison of effectiveness between IA and PLEX, and further randomised controlled trials are needed in this regard.

## Introduction

Chronic inflammatory demyelinating polyneuropathy (CIDP) is an autoimmune-inflammatory disease, which affects the myelin sheaths of peripheral nerves. While the typical clinical phenotype shows symmetrical distribution of motor and (in >50%) sensory symptoms, atypical variants like the distal acquired demyelinating symmetric syndrome (DADS), the multifocal acquired demyelinating sensory motor neuropathy (MADSAM), as well as pure motor and pure sensory forms have been described.^1^ Pathogenetically, autoimmune antibodies play an important role, as demonstrated by the efficacy of B-cell- and antibody-depleting therapies, as well as evidence of immunoglobulin G4 (IgG4) antibodies against structures of the node of Ranvier and adjacent regions in a subset of patients,^2^ which, meanwhile, have been classified as a distinct disease category.^1^

Therapy of CIDP includes treatment with steroids (evidence class Ib), intravenous immunoglobulins (IVIGs, Ia), plasma exchange (PLEX, Ib), and, since recently, efgartigimod.^3^ In some therapy-refractory cases, immunosuppressive drugs such as azathioprine and rituximab are used to lower steroid dosages or prolong treatment intervals. However, despite these treatment options, a significant share of patients still shows a progressive course of disease.^4^

Immunoadsorption (IA) constitutes an effective alternative to PLEX to remove autoimmune antibodies from the blood. While in PLEX, plasma is replaced by a substitution fluid, IA uses selective adsorber systems, which bind human antibodies and largely preserve other plasma proteins.^5^ Therefore, the processed plasma is reinfused to the patient, and no substitution fluids are needed. Since coagulation factors are spared, IA allows processing of higher plasma volumes per treatment and higher treatment frequencies compared to PLEX, resulting in greater reduction rates of IgG. For the same reason, adverse events seen in PLEX caused by loss of plasma proteins (like bleeding complications due to the loss of coagulation factors)^6^ are rare in IA.^7^ Therefore, IA is generally considered as a low-risk therapy.^8^ Of note, besides removal of auto-antibodies, further proposed mechanisms of action of IA include induction of autoantibody redistribution and subsequent immunomodulatory changes.^9^

Despite these promising prerequisites, evidence for IA in CIDP is low and mainly based on case series^10^, ^11^, ^12^ and one small randomised controlled study vs. PLEX (n = 20), which suggested at least similar efficacy. Moreover, long-term effects of repeated IA cycles on disease progression in CIDP have never been investigated; consequently, PLEX and IA are mainly regarded as rescue therapies rather than long-term treatment alternatives. Possible reasons for lacking evidence for IA include: (1) apheresis represents an interdisciplinary field, implying logistic challenges, (2) necessity of specialised infrastructure, limiting access to IA, (3) approval of medical devices (as opposed to drugs) does not necessitate conduction of large phase 3 clinical trials, and (4) placebo-controlled studies in IA are difficult to accomplish due to the invasiveness of the procedure.

Therefore, in this study, we sought to investigate efficacy and safety of IA in CIDP in a larger sample size, including evaluation of repeated IA cycles over a prolonged time period. For this purpose, between 2013 and 2025, we prospectively included 74 patients receiving IA, who had insufficiently responded to corticosteroids and/or immunoglobulins. We systematically recorded clinical scores, side effects, and laboratory data, and compared them with 25 refractory patients receiving PLEX. Moreover, we compared disease progression rates under repeated IA cycles with preceding therapies.

## Methods

### Study population and design

The study was conducted as an investigator-initiated trial at the Department of Neurology at Ulm University. Over a time period of >11 years (02/12/2013 until 03/03/2025), 80 patients were included in the study. All patients fulfilled the diagnostic criteria of typical CIDP according to the European Academy of Neurology/Peripheral Nerve Society (EAN/PNS) guideline on diagnosis and treatment of CIDP,^1^ had a continuously progressive course of disease, and had previously received treatment with steroids, IVIGs, or both, with insufficient response. Standard differential diagnosis included electrophysiology, nerve ultrasound, cerebrospinal fluid (CSF), vitamin and vasculitis screening, nodal/paranodal and antineural antibodies, hematology, tumor screening, and (in most patients) genetic testing for hereditary polyneuropathies. Patients were followed up until their last treatment of IA/PLEX or until the end of the observation period (02/2025). As this was an observational, non-randomised study, treatment decisions were made by treating physicians and respective patients in a process of shared decision making, including factors like pathophysiological considerations, accompanying diseases, and availability of treatment options.

### Ethics

The study was done in accordance with the Declaration of Helsinki, International Conference on Harmonization Guideline for Good Clinical Practice, and the applicable regulations. The Competent Ethics Committee of Ulm University, Germany, approved the study protocol (approval number 73/22). The study was conducted in adherence to standard guidelines (STROBE). All study participants gave their informed consent.

### Procedures

Before each treatment cycle of IA or PLEX, systemic infections were excluded by analyses of blood (leukocytes and CRP) and urine. Venous access was established by a central venous catheter (Shaldon catheter) which was placed in the right jugular vein by default. For long-term treatment, an arteriovenous shunt was established in 10/50 (20%) patients. This was done to facilitate venous access, in particular when placement of Shaldon catheters became increasingly difficult due to worsening status of the jugular veins.

Heparin and citrate were used as anticoagulants. Protein and, if necessary, potassium, were orally substituted during each cycle. Since there is no universally accepted standard regarding treatment regimens, all specifications such as number of treatments per cycle, treatment plasma volumes, and intervals between cycles were based on local experience and controlled by reduction rates of antibodies. One cycle of IA or PLEX consisted of 5 treatments on 5 consecutive days. The total individual plasma volume of each patient was calculated based on body weight, height, and hematocrit.

During IA, the 2-fold total plasma volume was processed on day 1, and the 2.5-fold total plasma volumes were processed on day 2–5, respectively. Two different adsorber systems were used: (1) ADAsorb (medicap clinic GmbH, Ulrichstein, Germany), containing to regenerable adsorbers with protein A (Immunosorba, Fresenius Medical Care, Bad Homburg, Germany) or the synthetic ligand peptide-GAM146 (Globaffin, Fresenius Medical Care, Bad Homburg, Germany); (2) TheraSorb, containing regenerable adsorbers with sheep IgG (Miltenyi Biotec, Bergisch Gladbach, Germany). During PLEX, the 0.7-fold total plasma volumes were removed each day using the cell separator COM.TEC (Fresenius Kabi AG, Bad Homburg, Germany) and substituted by 5% human albumin solution. These regimens were effective and well tolerated in previous trials.^10^^,^^13^ Although higher treatment volumes are commonly applied during PLEX in other neurological indications,^14^ the applied regimen was effective and well tolerated in a randomised controlled trial in multiple sclerosis^13^ as well as a previous CIDP trial.^10^

Time intervals between IA/PLEX cycles were individually determined based on treatment response, i.e., initial follow-up cycles were performed when patients noticed clinical worsening, and regular time intervals were set based on this assessment and later adjusted, if necessary. In cases of unsatisfactory treatment response, a switch from IA to PLEX or vice versa was considered.

### Outcome parameters

Clinical outcome measures were collected three days before the first treatment and after the last treatment of each cycle. As primary outcome measure, the CIDP score^10^ was used, which combines 3 standardised scales: (1) The Inflammatory Neuropathy Cause and Treatment (INCAT) disability score^15^ constitutes the standard clinical score for CIDP and consists of 2 items evaluating arm and leg function; (2) the Oxford muscle strength grading scale (Medical Research Council, MRC) as the standard scale for quantification of muscle strength between 0/5 (no movement) and 5/5 (full strength) at 32 pre-defined muscles (4 proximal and 4 distal muscles at each extremity); (3) vibration sensitivity testing with a 256-Hz tuning fork^16^ on a scale between 0/8 (no perception) and 8/8 (normal perception) at processus styloideus radii and malleolus lateralis on each side. The overall CIDP score ranges from 0 to 480 with each scale equally weighted (0–160). Compared to INCAT alone, the CIDP score has the advantage that it incorporates sensory signs and the MRC to measure smaller motor changes, which are not reflected by INCAT as a rough estimate of disability. In randomised controlled trials with new drugs focusing on patients in early disease stages, the INCAT alone constitutes a logical primary outcome measure as the goal of such studies is usually to show a meaningful clinical short-term improvement. Here, however, our main goal was to prevent or alleviate further disease progression in therapy-refractory patients, who predominantly featured long disease courses and prominent secondary axonal damage. As large improvements cannot be expected in this population, we decided to include two other well validated clinical scales.

Safety endpoints included terms and frequency of reported adverse events and serious adverse events, safety laboratory parameters (clinical chemistry and hematology), and vital signs. Values for safety laboratory parameters (blood count, electrolytes, liver and kidney values, and CRP) were compared with appropriate normal ranges and continuously monitored during each treatment cycle on a daily basis. All patients underwent continuous monitoring of vital signs (heart rate, blood pressure, and oxygen saturation) during apheresis. Reduction rates of immunoglobulins were calculated by comparing respective serum values before the first and after the last treatment of each cycle.

### Statistics

The sample size of this study was based on feasibility, i.e., to include as many patients as possible in a monocenter setting within the observation period, aiming at exceeding the number derived from a power analysis based on a previous study of IA in CIDP, using CIDP score as primary endpoint.^10^ In this previous study, median progression rates (CIDP score lost per month) were 0.15 for IA vs. 2.7 points for IVIG/MP, respectively. Based on these assumptions and using a two-sided type 1 error of 5% and a power of 80%, the sample size calculation suggested that the total number of required pairs (pre vs. post IA) is n = 35, which was exceeded by the number of patients actually included during the observation period.

Short-term effects were analysed by comparing absolute values of CIDP scores before and after each treatment cycle for both IA and PLEX. To evaluate long-term effects in the subgroup of patients receiving 2 or more cycles of IA or PLEX, we calculated progression rates per month based on the changes of CIDP scores as follows:

a) Pre-baseline progression rates were calculated by subtracting the absolute CIDP score at baseline from the maximum value (480), divided by the number of months between disease onset and baseline:

*Progression rate pre-baseline = (480 − CIDP score at baseline)/months between onset and baseline*

b) Likewise, progression rates during IA/PLEX were calculated by subtracting the CIDP score after the last treatment from the CIDP score before the first treatment (=baseline), divided by the number of months between first and last treatment:

*Progression rate during treatment = (CIDP score at baseline − CIDP score after last treatment)/months between first and last treatment*

All continuous data are given as median and interquartile range (IQR) or mean and standard deviation as appropriate. Categorical data are presented as frequencies and percentages. Changes in continuous data were investigated with the Wilcoxon signed rank test. Group comparisons for continuous data were performed using the Mann-Whitney-U-test or two-sample t-test as appropriate. Group comparisons for categorical data were carried out with the chi-square test or Fisher's exact test as appropriate. Adverse events were analysed descriptively. Missing data and missing follow-ups were not replaced.

Statistical analyses were done using GraphPad Prism, version 10.4.1.

### Role of the funding source

This work was an investigator-initiated trial without any institutional or industrial funding. Authors had full access to the data in the study. JD and ZE had the final responsibility for the decision to submit the manuscript for publication.

## Results

### Baseline characteristics

**Fig. 1:**
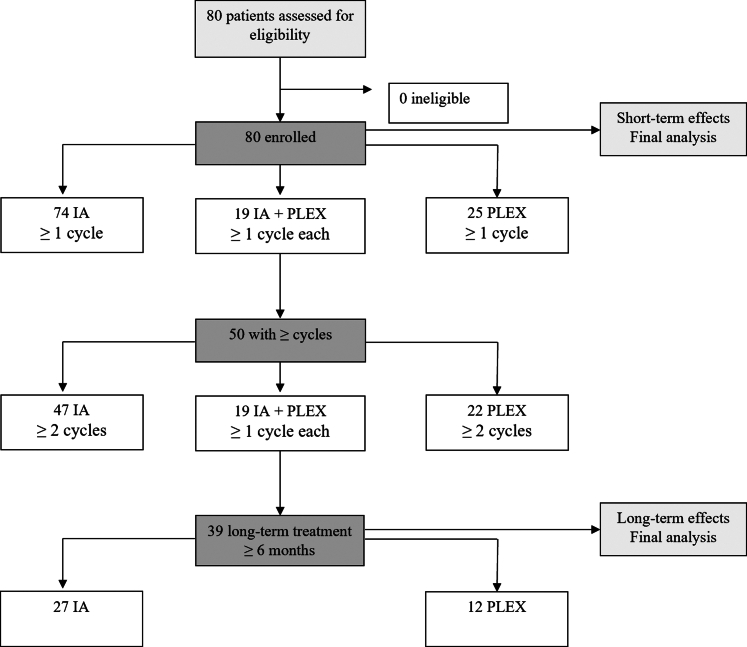
**Study flow diagram**. Discrepancies between the sum of immunoadsorption (IA) and plasma exchange (PLEX) subgroups and the overall number of patients in each row is due to patients receiving both IA and PLEX.

Between 12/2023 and 02/2025, 426 cycles (2130 treatment sessions) were performed in 80 patients. IA was applied in 74 patients (324 cycles, 1620 treatments), and PLEX in 25 patients (102 cycles, 510 treatments). The discrepancy between the sum of IA and PLEX subgroups (74 + 25 = 99) and the overall number of patients (80) is caused by the fact that 19 patients received both IA and PLEX (Fig. 1). Regarding baseline characteristics (demographic/clinical data and treatment regimens, Table 1), there were no significant differences between both treatment groups. Concomitant treatment with immunosuppressive agents was present in 14 (18.9%) IA and 5 (20%) PLEX patients. Paranodal antibodies (NF-155) were detected in one patient (IA #6 in Supplementary Table S1).

**Table 1:** Baseline characteristics.

	IA (n = 74)	PLEX (n = 25)	Total (n = 80)	p (IA vs. PLEX)
**Treatment cycles**	324	102	426	
**Patients with ≥2 cycles**	47 (63.5%)	22 (88.0%)	50	0.49
No. of cycles	4.0 (2.0–8.0)	4.0 (2.3–7.8)	4 (2–7.5)	0.95
Intervals between cycles	2.5 (1.6–5.5)	2.7 (2.1–3.7)	2.5 (1.9–4.3)	0.83
Total Treatment Time (months)	10.0 (5.5–38.0)	14.5 (6.3–22.8)	12.0 (6.0–34.0)	0.98
**Venous access**				
Shaldon	240 (74.1%)	79 (77.5%)	319 (74.9%)	0.52
Shunt	84 (25.9%)	23 (22.5%)	107 (25.1%)	
**Age**				
First treatment	64.1 ± 10.8	65.2 ± 10.7	64.4 ± 10.7	0.66
Last treatment	65.5 ± 10.4	66.1 ± 10.4	66.1 ± 10.2	0.80
**Sex**				
Male	59 (79.7%)	21 (84.0%)	64 (80.0%)	0.77
Female	15 (30.2%)	4 (16.0%)	16 (20.0%)	
**Disease duration** (months)	40.0 (18.0–82.5)	41.0 (10.5–74.0)	40.0 (15.8–80.5)	0.41
**CIDP score** (baseline)	330.0 (268.8–391.0)	309.0 (235.0–376.0)	328.0 (269.0–388.0)	0.26
**Progression rate pre-baseline** (CIDP score/month)	3.8 (2.2–9.1)	4.2 (2.6–17.2)	4.2 (2.5–12.5)	0.52
**Preceding therapies**				
IVIGs	60 (81.1%)	23 (92.0%)	65 (81.3%)	0.09
Corticosteroids	53 (71.6%)	14 (56.0%)	55 (68.8%)	0.22
**Immunosuppressants**	14 (18.9%)	5 (20.0%)	14 (17.5%)	>0.99
AZA	5 (6.8%)	1 (4.0%)	5 (6.3%)	
RTX	5 (6.8%)	3 (1.2%)	5 (6.3%)	
MMF	3 (4.1%)	1 (4.0%)	3 (3.8%)	
MTX	1 (1.4%)	0	1 (1.3%)	

Values are mean ± standard deviation, median (IQR), or n (%).

CIDP: Chronic inflammatory demyelinating polyneuropathy; IA: immunoadsorption; PLEX: plasma exchange; IVIGs: intravenous immunoglobulins; AZA: azathioprine; RTX: rituximab; MMF: mycophenolate mofetil; MTX: methotrexate.

### Short-term efficacy

**Fig. 2:**
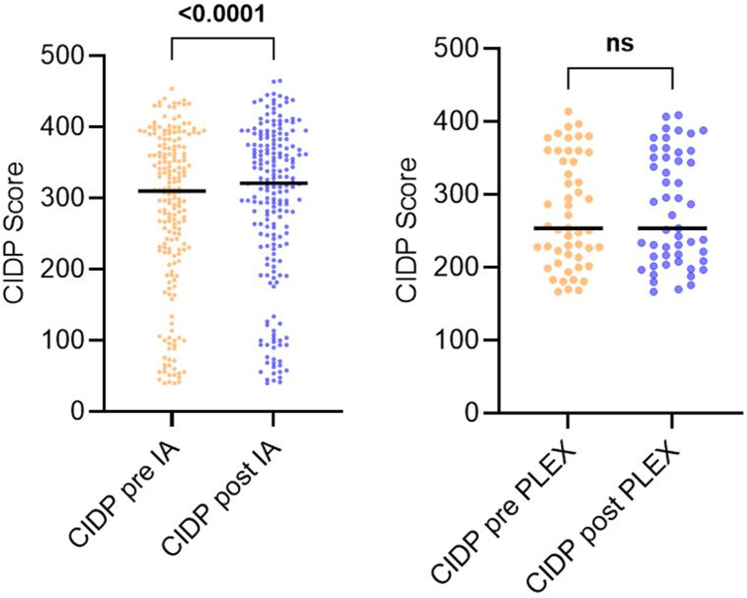
**Short-term effects of IA and PLEX**. Individual values for CIDP scores before (orange) and after (blue) each treatment cycle of IA (left) and PLEX (right). One cycle consists of 5 treatments. Data were collected 3 days before the first and directly after the last treatment of each cycle. CIDP: chronic inflammatory demyelinating polyneuropathy; IA: immunoadsorption; PLEX: plasma exchange.

Regarding short-term effects, IA patients showed an improvement of CIDP score from 310 (224–373) before IA to 321 (233–376) points directly after the last IA session of each cycle (median difference 5.0 (95% CI 2.0–6.0), p < 0.0001), while PLEX patients did not improve significantly (from 254 (214–358) before PLEX to 254 (209–351) after PLEX; median difference 0.0 (95% CI 0.0–5.0), Fig. 2). An improvement after the last treatment was found in 61.2% of IA cycles, but only in 47.1% of PLEX cycles (IA vs. PLEX p = 0.08). However, it has to be noted that follow-up visits might have occurred too early to capture the full effect.

### Long-term efficacy

The main goal of this study was to evaluate the long-term effects of repeated cycles of apheresis. Overall, 50 patients received 2 or more cycles (median 4 (2–7.5), maximum 43) over a median time period of 12 (6–34, maximum 123) months in median time intervals of 2.5 (1.9–4.3) months, of which 47 received at least one cycle of IA, and 22 received at least one cycle of PLEX. 19 patients received at least one cycle of both IA and PLEX due to unsatisfactory treatment response: 14 switched from IA to PLEX, of which 5 later switched back to IA, and 5 patients switched from PLEX to IA, of which 1 later switched back to PLEX. Among these patients, long-term effects were evaluated in all patients with an observation period of at least 6 months under the same therapy (n = 39, 27 patients receiving IA and 12 patients receiving PLEX).

**Fig. 3:**
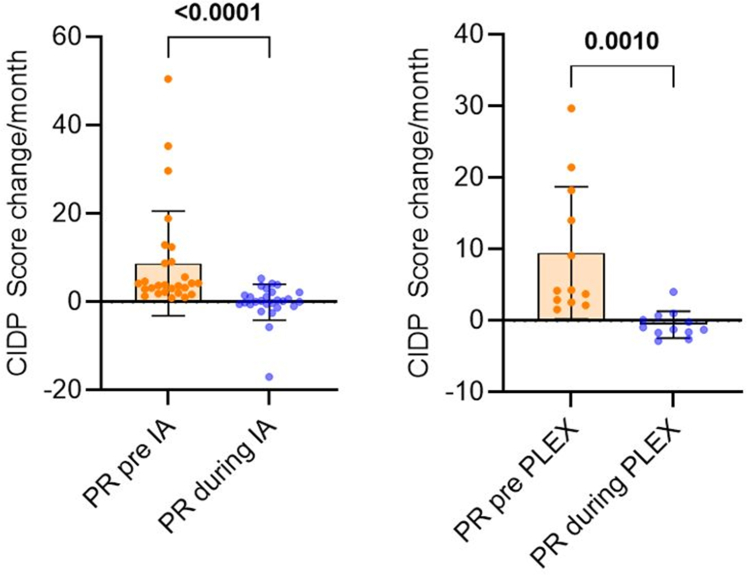
**Long-term effects of IA and PLEX**. Individual values for changes of CIDP score per month (blue) compared to preceding treatments with intravenous immunoglobulins and/or corticosteroids (orange) in patients receiving at last 2 cycles of IA (left) or PLEX (right). Negative values represent improvements of CIDP score. CIDP: chronic inflammatory demyelinating polyneuropathy; IA: immunoadsorption; PLEX: plasma exchange.

Compared to the unsuccessful preceding therapies with IVIGs and/or corticosteroids, IA patients showed a median improvement of progression rates from 3.83 (2.23–9.11) to 0.15 (−0.55 to 2.18) points of CIDP score lost per month (median difference −4.0 (95% CI −6.9 to −1.9), p < 0.0001), while in PLEX, progression rate improved from 4.21 (2.63–17.17) to −1.10 (−1.67 to 0.54; median difference −6.3 (95% CI −19.9 to −1.9), p = 0.0010; Fig. 3). Thus, IA patients displayed an almost complete stabilization of disease progression, while PLEX patients even showed a median improvement during the observation period. However, progression rates under treatment were not significantly different between IA and PLEX (p = 0.12). On an individual level, 25/27 patients receiving IA (92.6%) and 12/12 patients receiving PLEX (100%) showed an improvement of progression rates compared to preceding therapies.

Individual sub-score values (INCAT, MRC, and vibration testing) are given in Supplementary Table S1. Regarding INCAT, median values in both IA (6.0 (4.0–8.0) vs. 6.0 (5.0–7.0), p = 0.15) and PLEX (6.0 (5.0–8.0) vs. 6.0 (5.3–8.5), p > 0.99) did not change during the observation period. 11/27 (40.7%) patients in the IA group showed an improvement of 1 point or more, 11/27 (40.7%) were stable, and 5/27 (18.6%) worsened, while in PLEX, 3/12 (25.0%) improved, 7/12 (58.3%) were stable, and 2/12 (16.7%) worsened. The difference between IA and PLEX regarding responders and non-responders was not statistically significant (p = 0.48). Of note, the patient with NF-155 antibodies showed an excellent treatment response (INCAT +3 during the observation period).

MRC showed a slight, non-significant median improvement in both IA (148.0 (126.0–153.0) vs. 152.0 (123.0–155.0), p = 0.74) and PLEX (142.5 (125.5–130.0) vs. 151.0 (130.0–158.5), p = 0.29), while vibration sensitivity showed about stable median values in both IA (16.0 (11.0–24.0) vs. 16.0 (7.0–20.0), p = 0.26) and PLEX (13.0 (8.3–16.8) vs. 12.5 (6.3–16.8), p = 0.80).

### Side effects

Side effects per cycle (one cycle consisting of 5 treatments) are shown in Table 2. Overall, both IA and PLEX were well tolerated. Regarding overall frequency and severity of side effects, there were no striking differences between IA and PLEX. Procedure-specific side effects were rare and mild, with infections representing the most common side effect in 2.3% of cycles (2.2% in IA and 2.9% in PLEX).

**Table 2:** Side effects.

	IA (n = 74)	PLEX (n = 25)	Total (n = 80)
**Treatment cycles**	324	102	426
**General**			
Bradycardia	1 (0.3%)	0	1 (0.2%)
Hypotonia	2 (0.6%)	0	2 (0.5%)
Exanthema	1 (0.3%)	1 (1.0%)	2 (0.5%)
Infection	7 (2.2%)	3 (2.9%)	10 (2.3%)
Gastrointestinal	1 (0.3%)	0	1 (0.2%)
Urinary tract	4 (1.2%)	3 (2.9%)	7 (1.6%)
Respiratory	1 (0.3%)	0	1 (0.2%)
Unknown	1 (0.3%)	0	1 (0.2%)
Technical Defects	1 (0.3%)	0	1 (0.2%)
**Shaldon-associated**	240 cycles	79 cycles	319 cycles
Thrombosis of jugular vein	16 (6.7%)	6 (7.6%)	22 (6.9%)
Symptomatic	3 (1.3%)	0	3 (0.9%)
Asymptomatic	13 (5.4%)	6 (7.6%)	19 (6.0%)
Bleeding/Hematoma	1 (0.4%)	2 (2.5%)	3 (0.9%)
Dislocation	3 (1.3%)	1 (1.2%)	3 (0.9%)
Sepsis (focus Shaldon)	1 (0.4%)	0	1 (0.3%)
Pneumothorax	1 (0.4%)	0	1 (0.3%)
**Shunt-associated**	84	23	107
Occlusion	7 (8.3%)	0	7 (6.5%)
Bleeding/Hematoma	2 (2.4%)	1 (4.3%)	3 (2.8%)
Radialis Lesion (surgery)	1 (1.2%)	0	1 (0.9%)

Side effects on per-cycle basis (1 cycle consisting of 5 treatments) in 324 cycles of IA and 102 cycles of PLEX. Side effects due to venous access are related to the number of cycles performed with Shaldon (n = 240) or shunt (n = 79), respectively.

IA: immunoadsorption; PLEX: plasma exchange.

However, side effects related to venous access (Shaldon catheter or intravenous shunt) were more frequent and significant. Most importantly, thromboses of the jugular vein were detected by ultrasound in 6.9% of cycles with Shaldon catheter (6.7% in IA and 7.6% in PLEX), of which 18.8% were symptomatic, causing pain and swelling. All jugular thromboses were treated with anticoagulants. Further complications included one pneumothorax and one sepsis due to catheter infection. All patients later recovered without persistent damage.

In patients with shunts, an occlusion occurred in 6.5% of cycles, which necessitated revision by balloon dilatation and/or surgery, and one patient suffered a radialis lesion during surgery, causing a persistent pain syndrome.

### Safety parameters

Laboratory changes during each cycle are shown in Table 3. These changes were all temporary and asymptomatic, except leukocytosis and increase of CRP during infections. All patients received prophylactic oral substitution of protein and potassium, and PLEX patients received albumin solution during each treatment. Of note, anemia was more common in PLEX (60.8% vs. 21.5%, p < 0.0001), while thrombopenia was more common in IA (33.6% vs. 4.9%, p < 0.0001). In some patients, renal retention parameters (urea and creatinine) temporarily increased during treatment (3.1%/5.2% in IA, 8.8%/2.0% in PLEX).

**Table 3:** Laboratory changes.

	IA (n = 74)	PLEX (n = 25)	Total (n = 80)
**Treatment cycles**	324	102	426
Leukocytosis	21 (5.6%)	10 (9.8%)	31 (7.3%)
Leukopenia	4 (1.2%)	0	4 (0.9%)
Anemia	70 (21.5%)	63 (60.8%)	133 (31.2%)
Thrombopenia	109 (33.6%)	5 (4.9%)	114 (26.8%)
Hypokalemia	35 (10.8%)^a^	0^a^	35 (8.2%)
Hyponatremia	0	2 (2.0%)	2 (0.5%)
Hypocalcemia	6 (8.1%)	3 (2.9%)	9 (2.1%)
Urea^b^	10 (3.1%)	9 (8.8%)	19 (4.5%)
Creatinine^b^	17 (5.2%)	2 (2.0%)	19 (4.5%)
Hypoproteinemia	324 (100%)^a^	96 (94.1%)^a^	420 (98.6%)
CRP^b^	51 (15.7%)	8 (7.8%)	59 (13.8%)

Laboratory changes on per-cycle basis (1 cycle consisting of 5 treatments) in 324 cycles of IA and 102 cycles of PLEX.

IA: immunoadsorption; PLEX: plasma exchange; CRP: C-reactive protein.

a Protein and potassium were orally substituted, and PLEX patients received albumin solution during each treatment.

b Elevated.

### Immunoglobulins

**Fig. 4:**
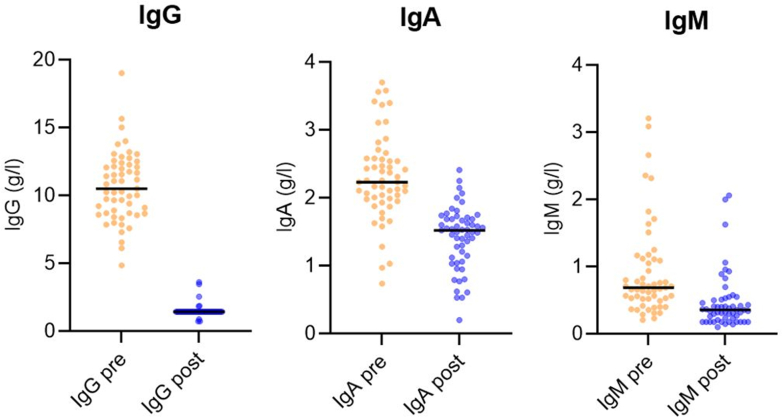
**Reduction rate of immunoglobulins**. Individual values of 55 cycles showing immunoglobulin levels of IgG (left), IgA (center), and IgM (right) after the last treatment of each cycle (post, blue) compared to baseline (before first treatment of each cycle, orange). Ig: Immunoglobulin.

Ig reduction rates for IA (measured directly after the last treatment and related to baseline before first treatment of each cycle) are depicted in Fig. 4 based on measurements in a subgroup of n = 55 cycles. Reduction rates were 85.7% for IgG, 38.3% for IgA, and 48.4% for IgM.

## Discussion

This study aimed at evaluating the therapeutic value of IA in CIDP. Currently, IA is mainly used as a rescue option in case of acute worsening in patients who respond insufficiently to other treatment options. However, there are several arguments that repeated IA might also be useful as a long-term therapeutic alternative. First, although most patients respond well to the first-line treatment options, i.e., IVIGs or corticosteroids, a smaller, but still meaningful share of patients shows a progressive course of disease under standard therapy.^4^ Second, it is well-known that immunoadsorption features excellent safety and tolerability as—contrary to PLEX—plasma proteins like coagulation factors are largely preserved.^7^^,^^8^^,^^17^ Third, autoantibodies play an important role in pathogenesis of CIDP, and efgartigimod, which has been recently approved,^3^ follows the same therapeutic principle by repeatedly reducing circulating antibodies to attenuate inflammation and prevent long-term axonal damage. However, as suggested by the efgartigimod trial as well as a retrospective case series in PLEX/IA,^12^ evidence of specific autoimmune antibodies does not seem to constitute a necessary prerequisite for efficacy of antibody-depleting treatments in CIDP, possibly because some disease-related antibodies remain still undetected and/or due to secondary immune-modulating effects of these therapies.

Despite these strong arguments, evidence for the use of IA in CIDP is low, mainly relying on one small randomised controlled study with 20 patients vs. PLEX,^18^ while long-term effects are largely unexplored. Therefore, we prospectively evaluated the course of disease of 80 patients receiving IA or PLEX over an observation period of >10 years. Importantly, all patients had been unsuccessfully treated with IVIGs and/or corticosteroids previously. Similar to the common practice with IVIGs, treatment intervals were individually modified during the observation period, i.e., subsequent cycles were performed when symptoms began to worsen again, which resulted in median treatment intervals of 2.5 months, although with great interindividual differences, mirroring heterogenous progression rates in this heterogenous disease.

We found that the course of disease was almost completely stabilised by both repeated IA and repeated PLEX, which was signified by a significant reduction of progression rates as measured by loss of CIDP score per month, which combines INCAT, MRC, and vibration sensitivity, including several patients who were treated regularly over several years without any additional comedication. Regarding long-term success, there were no significant differences between IA and PLEX. Regarding the incorporated subscales, we found that some patients showed improved motor function (MRC), while others showed improved sensitivity, which translated to improved INCAT in some, but not all of these patients. However, there were also therapy-refractory patients who continued to decline. The only previous study evaluating repeated cycles of IA in CIDP (although with 6 months significantly shorter) found greater response rates for IA compared to IVIGs, but concluded that this finding was probably biased as patients in the IA group were more severely affected at baseline.^19^

Moreover, as opposed to PLEX, we found that patients receiving IA also showed immediate benefits, i.e., CIDP scores improved directly after each cycle, although it must be mentioned that these short-term effects were relatively small. Regarding the missing short-term effect of PLEX, three possible explanations have to be taken into account: First, clinical evaluations might have occurred too early in order to capture the full therapeutic effect, which usually takes several days to fully develop. Second, the treated plasma volume per treatment was comparatively low (0.7-fold) compared to other studies; therefore, it is possible that higher plasma volumes might have yielded better results, but likely also a higher incidence of side effects. Third, most patients had a long course of disease, so irreversible axonal damage must be suspected—thus, the extent of potential short-term improvement is naturally limited. Since PLEX patients had lower median CIDP scores at baseline, this phenomenon might have been more pronounced in the PLEX group.

On the other hand, of note, the potential superiority of IA over PLEX regarding short-term effects are in line with the only randomised controlled study in 10 vs. 10 patients conducted by Lieker et al.,^18^ in which IA patients likewise showed a greater response rate (measured by INCAT and MRC) compared to PLEX directly after treatment. Another case series in therapy-refractory patients with CIDP found an even higher response rate of IA (13/14, 92.9%),^11^ while one recent retrospective study comparing 23 PLEX vs. 21 IA patients^20^ found about comparable short-term effects on INCAT. Regarding comparability of these IA studies, it has to be noted that different adsorber systems with different adsorption profiles (one-time tryptophan adsorbers) were used in the majority of above cited studies.

Regarding safety, and also in line with the aforementioned study,^18^ procedure-specific side effects were rare and mild in both groups. This might seem surprising at first, because superior tolerability is generally regarded as the main advantage of IA over PLEX as significant adverse events like bleeding complications and severe allergic reactions related to substitution solutions have been frequently described in PLEX.^5^^,^^6^^,^^21^, ^22^, ^23^, ^24^, ^25^, ^26^ However, this finding might likely be explained by the comparatively low plasma volume treated per session during PLEX in this study, which allowed daily treatments while avoiding the use of fresh frozen plasma.

However, significant side effects were recorded in the context of venous access as a necessary requirement for apheresis. Most importantly, we found a significant incidence (6.9%) of thromboses of the respective jugular vein caused by the Shaldon catheter. Although these were asymptomatic in most cases, deep venous thromboses require treatment with anticoagulants, which in turn increase the risk of bleeding complications. Moreover, one pneumothorax and one catheter-associated sepsis occurred in one patient, respectively. Surgically applied arteriovenous shunts were used as an alternative venous access in some patients receiving long-term treatments, but these did not represent a flawless solution either, as occlusions necessitating surgical revision occurred frequently (6.5%). As another option, peripheral access could be considered in patients with appropriate venous status; however, this implies significant burden for patients as both arms are immobilised for several hours during each treatment, and accidental movements might cause technical failure. In summary, establishing stable venous access for long-term, repeated apheresis remains as challenge, which has not been solved in a satisfactory manner to date and constitutes a limiting factor for long-term applicability of apheresis in CIDP.

Still, although IA should not be considered a first-line treatment option due to its invasiveness, study results show that application of repeated IA constitutes a promising treatment option in patients who insufficiently respond to IVIGs and corticosteroids and might be more effective and/or less risky (depending on treated plasma volumes) compared to PLEX, although a definite conclusion can still not be drawn due to the lack of a sufficiently powered randomised controlled study comparing both options; however, such a study evaluating long-term outcome parameters will be difficult to conduct. Likewise, to date, it is unclear, whether repeated IA is superior or inferior to efgartigimod, which relies on a similar mode of action, as comparative studies are missing. Of note, treatment intervals (median 2.5 months) were significantly longer as commonly applied for IVIGs, offering advantages from an economic and logistic point of view, and partly compensating for its invasiveness regarding burden for patients.

The long observation period, the comparatively high number of patients compared to other studies, the existence of a control group receiving PLEX, and the prospective evaluation of standardised clinical outcome measures and side effects represent the main strengths of this studies. Regarding limitations, it must be acknowledged that group allocation was not randomised and that data related to preceding treatments were collected retrospectively, the latter implying the risk of inaccurate information including the simplified assumption of a linear worsening pre-IA, and possibly also sub-optimal treatment regimens prior to baseline. Although the CIDP score consists of three well-validated scales, validity and reliability of the combined score has not yet been determined independently. Moreover, the observational study design with a long observation time implied that patient numbers were limited and that interfering elements (such as cross-overs between IA and PLEX, effect of aging, placebo and nocebo effects) would occur. However, these limitations were deliberately accepted to facilitate the exceptional longevity of the study.

## Contributors

Conceptualization: JD; Data Curation: JD, ZE, KK, OS, LJ; Formal Analysis: JD, ZE, BM; Investigation: JD, ZE, CH, KK, OS, VR, LJ, LR, MW, TF, SJ, RG, AR, MS, HT; Methodology: JD; Project Administration: JD; Resources: JD; Supervision: JD; Visualization: JD, ZE, CH; Writing–original draft: JD, ZE; Writing–review and editing: JD, ZE, CH, KK, OD, LJ, LR, MW, TF, SJ, RG, AR, MS, HT, JHW, BM. JD and ZE have directly accessed and verified the underlying data reported in the manuscript. All authors confirm that they read and approved the final version of the manuscript.

## Data sharing statement

Individual participant data that underlie the results reported in this article, after de-identification (text, tables, and figures) will be available. Data will be available beginning 3 months and ending 5 years following article publication. Data will be shared with researchers who provide a methodologically sound proposal. Data will be shared for analyses to achieve the aims in the approved proposal. Proposals should be directed to johannes.dorst@uni-ulm.de; to gain access, data requestors will need to sign a data access agreement. Data are available for 5 years at https://www.uniklinik-ulm.de/neurologie.html.

## Declaration of interests

JD reports research grants from Fresenius Medical Care GmbH, Fresenius Medical Care Deutschland GmbH, Miltenyi Biotec, and Diamed GmbH, as well as personal fees from Fresenius Medical Care GmbH, Fresenius Medical Care Deutschland GmbH, Miltenyi Biotec, Diamed GmbH, and argenx. ZE reports personal fees from Fresenius Medical Care GmbH. HT reports research and sponsoring grants from Fresenius Medical Care GmbH and Fresenius Medical Care Deutschland GmbH. MS has received honoraria for speaking and/or travel grants from Bayer, Biogen and TEVA and research funding from the Hertha-Nathorff-Program and University of Ulm, none related to this study. AR reports research grants from Deutsche Gesellschaft für Muskelkranke e. V. as well as personal fees from Amicus, Sanofi, Sun Pharmaceuticals, Argenx, Fulcrum, Alexion, outside the submitted work. CH, KK, OS, VR, LJ, LR, MW, TF, SJ, RG, JHW, and BM declare no competing interests.

## Appendix A Supplementary data

Supplementary Material

Translated Abstract
