# Supplementary material for: Evaluating the short-term and long-term therapeutic effects of immunoadsorption compared with plasma exchange in chronic inflammatory demyelinating polyneuropathy: a long-term, prospective, observational study
Source: eClinicalMedicine. 2026 Jan 8;91:103742. doi: 10.1016/j.eclinm.2025.103742 (PMC12818062; doi:10.1016/j.eclinm.2025.103742)
Supplement: Supplementary Material [file mmc1.pdf]

Study Protocol:
Repeated Immunoadsorption for CIDP -
A Long-Term Observational Study (RICO)

**TITLE OF STUDY**	**Repeated Immunoadsorption for CIDP - A Long-Term Observational Study**
**SHORT TITLE**	**RICO**
**PROTOCOL NUMBER**	**RICO-1.0**
**CONDITION**	Chronic Inflammatory Demyelinating Polyneuropathy (CIDP)
**STUDY TYPE/ OVERVIEW**	Observational, prospective, monocenter study.<br><br>This study is designed as an explorative study aiming at exploring safety and efficacy of repeated immunoadsorption (IA) in patients with Chronic Inflammatory Demyelinating Polyneuropathy (CIDP) compared to plasma exchange (PLEX) in patients who show a progressive course of disease under intravenous immunoglobulins (IVIG), methyl-prednisolone (MP), or both. For this purpose, patients receiving their first cycle of IA or PLEX will be included an followed up in 6-month intervals until the end of IA/PLEX treatment or until the study will be terminated.
**SUMMARY/BACKGROUND**	Chronic inflammatory demyelinating polyneuropathy (CIDP) is a rare autoimmune-inflammatory disease which mainly affects the myelin sheaths of peripheral nerves, leading to paresis and sensory deficits. Clinical presentation varies greatly among patients. While the typical clinical phenotype shows symmetrical distribution of motor and (in >50%) sensory symptoms, about half of the patients display variants with pure motor or pure sensory deficits as well as asymmetric manifestations. The course of disease is chronically progressive in most cases but can be relapsing as well.<br><br>Therapy of CIDP includes treatment with IVIg (evidence class Ia), MP (Ib), plasma exchange (PE; Ib), and various immunosuppressive drugs (IV). Individual response to different options varies greatly among patients, and no unanimously accepted guidelines or therapeutic schemes exist. The European Federation of Neurological Societies (EFNS) guidelines recommend to begin with IVIg or MP as non-invasive options which are easy to apply, and to use PE in case of insufficient therapeutic response[1]. Long-term immunosuppressive agents are used if a long-lasting effect cannot be achieved with the above-mentioned therapies or in order to lower MP dosages. Current guidelines for CIDP assume an about equal efficacy of IVIg, MP, and PE, based on current evidence[2]. In summary, therapy of CIDP is rather pragmatic than specific, and in many cases different therapeutic schemes have to be tried before an effective approach is found for the individual patient. Moreover, a significant share of patients do not respond to first-line treatment options[3].<br><br>IA is a highly effective and well tolerated method to remove autoantibodies from the blood. Further proposed mechanisms of action include induction of autoantibody redistribution and subsequent immunomodulatory changes[4]. During the procedure, the patient's plasma runs through adsorber systems which selectively bind human immunoglobulins before it is returned to the patient. Therefore, in contrast to PLEX, all other plasma proteins like coagulation factors are largely preserved[5], allowing higher treatment frequencies and higher plasma volumes (PVs) to be processed. Adverse events in PLEX, which are based on the loss of plasma proteins (like

	bleeding complications due to the loss of coagulation factors)[6], are rare in IA, which is generally considered as a low-risk therapy[7]. Furthermore, no substitution solutions like human albumin solutions or fresh frozen plasma are needed. Thus, better tolerability of IA compared to PLEX is regarded as one of the main advantages of IA and has been demonstrated in clinical studies[8].
**RESEARCH QUESTION AND OBJECTIVES**	The objective of this trial is to investigate whether IA constitutes an effective and safe escalating therapeutic option in CIDP. For this purpose, IA will be performed in patients who showed insufficient therapeutic response to IVIg and/or MP, and progression rates under therapy will be compared before and after the switch to IA as well as with a control cohort of patients receiving PLEX. Moreover, short-time effects of IA and PLEX will be evaluated by comparing clinical scores before and after each cycle.<br><br>One previous study in 20 patients[8] found a higher response rate for patients treated with IA compared to PLEX (66.7% vs. 44.4%) after a follow-up of 4 weeks. Another non-randomized trial found that patients with therapy-refractory progressive disease courses could be stabilized by periodical cycles of IA[9]. However, in summary, evidence for the use of IA in CIDP is low, especially since there are no studies investigating the mid-term and long-term effects of repeated IA compared to other treatment options, and most studies refer to small sample sizes.<br><br>Therefore, in this study, we seek to evaluate safety and efficacy of IA in therapy-refractory CIDP in a prospective study design over a prolonged period of time, applying periodical cycles of therapies. Various standardized primary and secondary efficacy endpoints will be collected.<br><br>**Objectives:**<br>(1) to investigate efficacy of repeated IA measured by CIDP score[9] and various other standardized efficacy parameters compared to a preceding IVIG and/or MP therapy.<br>(2) to investigate efficacy and safety of repeated IA compared to repeated PLEX<br>(3) to investigate the effect of IA on various additional secondary endpoints, such as standard laboratory parameters and immunoglobulins
**TRIAL DURATION/ SCHEDULE**	As the main goal of the trial is to obtain long-term efficacy and safety data, the exact observation period of the trial is not defined. The trial is expected to last several years, and repeated analysis of data will be conducted about every 10 years.<br>Before their first cycle of IA/PLEX, patients will perform a baseline visit. Regular follow-up visits will take place every 6 months. The schedule of study examinations is depicted in the synopsis below. During the observation period, patients switching from IA to PLEX or vice versa will remain in the study, while patients ending IA/PLEX treatment will end the study. Recruitment is expected to start in 12/2013, thus the anticipated date of first analysis of data is 12/2023.
**TREATMENT GROUPS**	For IA, the following therapeutic scheme will be applied:<br>- 5 treatments on 5 consecutive days, 2–2.5-fold individual PV per treatment<br>- treatment intervals: 2–24-week intervals, according to individual response and duration of treatment effects.<br><br>For PLEX, the following therapeutic scheme will be applied:<br>- 5 treatments on 5 consecutive days, 0.7-fold individual PV per treatment<br>- treatment intervals: 2–24-week intervals, according to individual response and duration of treatment effects.<br><br>The decision for IA or PLEX will be made by the patient in a process of shared decision making after detailed information by the treating physician about all available treatment options.

	Patients may receive concomitant immunosuppressive drugs, such as azathioprine or rituximab.
**NUMBER OF PATIENTS AND CENTERS**	As an explorative study in a rare disease, the number of patients will be based on feasibility, i.e., the ability to recruit patients within 10 years within a monocenter setting. We expect, that between 50-100 patients can be recruited within this time frame, which will be enough to test for efficacy of IA based on the statistical power calculation (see below).
**ENDPOINTS**	**Primary Outcome Measure**<br>CIDP Score, combining INCAT, MRC, and Vibration Sensitivity[9]<br><br>**Secondary Outcome Measures**<br>- Therapeutic Response (share of patients with improvement of CIDP score)<br>- Type and frequency of AEs and SAEs<br>- Type and frequency of abnormal laboratory findings, including blood count, electrolytes, liver and kidney values, CRP<br>- Immunoglobulin reduction rates (for patients receiving IA)
**INCLUSION AND EXCLUSION CRITERIA**	Inclusion criteria:<br>- Diagnosis of CIDP based on the EAN/PNS Guidelines on Diagnosis and Treatment of CIDP[2]<br>- age ≥ 18 years<br>- Willing and capable of giving written informed consent<br>- Previous unsuccessful treatment with IVIG or MP (as judged by the treating physician)<br>- Willing to receive their first cycle of IA or PLEX<br><br>Exclusion criteria: none
**STATISTICAL ANALYSIS**	**a) Sample size**<br>Due to the paired study design, i.e. comparison of pre- and post-IA treatment progression rates measured as change of CIDP score in the same patients, sample size estimation is based on a Wilcoxon signed rank test in order to address the non-normal shape of the CIDP distribution. Prior data[9] of the CIDP score comparing IA treatment with preceding IVIg/MP treatment accurately reflects the goal of our study. In this study, median progression rates (CIDP score lost per month) were 0.15 for IA vs. 2.7 points for IVIg/MP, respectively. Based on these assumptions and using a two-sided type 1 error of 5% and a power of 80%, the sample size calculation suggests that the total number of required pairs is n=35.<br><br>**b) Efficacy**<br>Pre-post comparison of progression rates before/after IA treatment measured as change in CIDP score, as well as all other continuous outcome parameters, will be analyzed by means of the Wilcoxon signed rank test. In case of further secondary, categorical variables, the McNemar test will be performed. Generally, 95% confidence intervals will be calculated for all point estimates. All continuous efficacy parameters will be measured as change from baseline and analyzed as change per month. Mixed linear regression models will be applied to account for correlated data. Additionally, patients receiving IA will be compared to patients receiving PLEX. All analyses will be performed on a two-sided significance level of 5%.<br><br>**c) Safety**<br>AEs and SAEs will be analyzed by displaying absolute and relative frequencies.

**STUDY INVESTIGATORS**	Prof. Dr. Johannes Dorst (PI)<br><br>Department of Neurology<br>University Clinic Ulm<br>Oberer Eselsberg 45<br>89081 Ulm / Germany<br>phone: +49 (0) 731 177 5285<br>fax: +49 (0) 731 177 1202<br>email: johannes.dorst@uni-ulm.de
**STUDY STATISTICIAN**	Prof. Dr. Benjamin Mayer<br>Department of Epidemiology and Medical Biometry<br>University Clinic Ulm

Appendix A - Study Schedule

Measure	<u>Inclusion</u> (V0)	<u>Follow-Up Visits</u> 6-monthly intervals (V1–V3)
Patient Information / Informed Consent *	x	
Medical History	x	
INCAT	x	x
MRC	x	x
Vibration Sensitivity	x	x
Adverse Events		x
Venous blood sampling	x	x

1. Van den Berg JP, Kalmijn S, Lindeman E, et al. Multidisciplinary ALS care improves quality of life in patients with ALS. *Neurology* 2005; **65**(8): 1264-7.
2. Van den Bergh PYK, van Doorn PA, Hadden RDM, et al. European Academy of Neurology/Peripheral Nerve Society guideline on diagnosis and treatment of chronic inflammatory demyelinating polyradiculoneuropathy: Report of a joint Task Force-Second revision. *European journal of neurology : the official journal of the European Federation of Neurological Societies* 2021; **28**(11): 3556-83.
3. Lehmann HC, Burke D, Kuwabara S. Chronic inflammatory demyelinating polyneuropathy: update on diagnosis, immunopathogenesis and treatment. *Journal of neurology, neurosurgery, and psychiatry* 2019; **90**(9): 981-7.
4. Klingel R, Heibges A, Fassbender C. Plasma exchange and immunoadsorption for autoimmune neurologic diseases - current guidelines and future perspectives. *Atherosclerosis Supplements* 2009; **10**(5): 129-32.
5. Zollner S, Pablik E, Druml W, Derfler K, Rees A, Biesenbach P. Fibrinogen reduction and bleeding complications in plasma exchange, immunoadsorption and a combination of the two. *Blood purification* 2014; **38**(2): 160-6.
6. Basic-Jukic N, Kes P, Glavas-Boras S, Brunetta B, Bubic-Filipi L, Puretic Z. Complications of therapeutic plasma exchange: experience with 4857 treatments. *Therapeutic apheresis and dialysis : official peer-reviewed journal of the International Society for Apheresis, the Japanese Society for Apheresis, the Japanese Society for Dialysis Therapy* 2005; **9**(5): 391-5.
7. Dorst J, Fillies F, Dreyhaupt J, Senel M, Tumani H. Safety and Tolerability of Plasma Exchange and Immunoadsorption in Neuroinflammatory Diseases. *J Clin Med* 2020; **9**(9).
8. Lieker I, Slowinski T, Harms L, Hahn K, Klehmet J. A prospective study comparing tryptophan immunoadsorption with therapeutic plasma exchange for the treatment of chronic inflammatory demyelinating polyneuropathy. *Journal of clinical apheresis* 2017; **32**(6): 486-93.
9. Dorst J, Ludolph AC, Senel M, Tumani H. Short-term and long-term effects of immunoadsorption in refractory chronic inflammatory demyelinating polyneuropathy: a prospective study in 17 patients. *Journal of neurology* 2018; **265**(12): 2906-15.

Supplementary Table 1: Sub-sores in patients receiving long-term treatment

	INCAT pre	INCAT post	MRC pre	MRC post	VIB pre	VIB post
**IA** (sex, age)						
**#1 (male, 55)**	9	10	152	155	16	16
**# 2 (male, 54)**	0	5	66	112	2	4
**# 3 (male, 57)**	4	7	112	120	11	13
**#4 (male, 63)**	8	7	148	160	24	19
**#5 (male, 58)**	9	9	149	147	21	17
**#6 (female, 51)**	2	5	82	101	2	0
**#7 (male, 78)**	6	6	152	157	16	18
**#8 (female, 68)**	5	6	129	160	12	20
**#9 (male, 67)**	5	5	144	125	18	17
**#10 (female, 75)**	7	8	153	152	30	30
**#11 (male, 55)**	6	7	152	148	4	4
**#12 (female, 62)**	4	5	156	152	16	18
**#13 (male, 71)**	8	8	142	144	23	16
**#14 (male, 77)**	8	7	158	152	9	7
**#15 (female, 68)**	5	5	135	119	7	6
**#16 (male, 63)**	7	7	160	154	14	22
**#17 (male, 58)**	4	4	126	123	24	24
**#18 (male, 52)**	5	2	24	20	6	4
**#19 (male, 56)**	4	6	126	164	21	0
**#20 (male, 58)**	8	7	144	144	25	27
**#21 (male, 63)**	9	6	99	61	26	7
**#22 (male, 72)**	7	7	153	152	14	14
**#23 (male, 81)**	5	5	152	158	26	17
**#24 (male, 64)**	6	6	154	157	13	7
**#25 (female, 77)**	4	4	144	144	11	10
**#26 (male, 43)**	7	9	154	155	25	32
**#27 (male, 55)**	8	10	157	154	26	21
**PLEX** (sex, age)						
**#1 (male 55)**	8	9	157	156	22	21
**#2 (male, 71)**	5	5	123	128	4	8

**#3 (male, 65)**	9	9	151	159	17	7
**#4 (male, 58)**	10	10	153	153	13	17
**#5 (female, 52)**	6	6	87	85	0	0
**#6 (male, 80)**	8	6	160	159	18	12
**#7 (male, 80)**	6	6	156	157	13	13
**#8 (female, 69)**	4	5	125	140	8	6
**#9 (male, 63)**	5	7	127	149	16	18
**#10 (male, 71)**	6	6	134	124	13	6
**#11 (male, 83)**	8	7	160	160	14	15
**#12 (female, 49)**	4	4	134	136	9	16

Subscores in patients receiving long-term treatment (>6 months) of repeated immunoadsorption (IA) or plasma exchange (PLEX). Individual values refer to baseline (before first treatment, "pre") and after last treatment ("post"). INCAT: Inflammatory Neuropathy Cause and Treatment disability score; MRC: Medical Research Council; VIB: vibration testing with tuning fork.
