# Supplementary material for: Evaluating the short-term and long-term therapeutic effects of immunoadsorption compared with plasma exchange in chronic inflammatory demyelinating polyneuropathy: a long-term, prospective, observational study
Source: eClinicalMedicine. 2026 Jan 8;91:103742. doi: 10.1016/j.eclinm.2025.103742 (PMC12818062; doi:10.1016/j.eclinm.2025.103742)
Supplement: Translated Abstract [file mmc2.docx]

*The following translations in German were submitted by the authors and we reproduce them as supplied. They have not been peer reviewed. Our editorial processes have only been applied to the original abstract in English, which should serve as reference for this manuscript.*

**Zusammenfassung**

**Hintergrund:** Die Chronische Inflammatorische Demyelinisierende Polyneuropathie (CIDP) ist eine autoimmun-entzündliche Erkrankung des peripheren Nervensystems. Kortikosteroide, intravenöse Immunglobuline (IVIGs) und Plasmaaustausch (plasma exchange, PLEX) repräsentieren die wesentlichen therapeutischen Möglichkeiten, jedoch stellt die Immunadsorption (IA) aufgrund ihrer hervorragenden Verträglichkeit und der Fähigkeit, höhere Raten von Autoimmunantikörpern zu entfernen, eine beachtenswerte Alternative zur PLEX dar. Allerdings ist die Evidenz hinsichtlich ihrer therapeutischen Wertigkeit bei CIDP gering, und der Effekt von wiederholter IA als Langzeittherapie ist weitgehend unbekannt. Diese Studie hatte daher zum Ziel, Kurz- und Langzeiteffekte der IA bei CIDP im Vergleich zur PLEX und vorangegangenen Therapien (IVIGs und Kortikosteroide) zu evaluieren.

**Methoden:** Zwischen dem 2.12.2023 und dem 3.3.2025 evaluierten wir prospektiv den Krankheitsverlauf von CIDP-Patienten, die mindestens einen Zyklus IA oder PLEX über Shaldon-Katheter oder arteriovenösen Shunt erhielten. Alle Patienten wurden in der Abteilung für Neurologie der Universität Ulm, Deutschland, behandelt. Alle Patienten erfüllten die diagnostischen Kriterien einer typischen CIDP entsprechend der Leitlinie der European Academy of Neurology/Peripheral Nerve Society (EAN/PNS) für Diagnose und Therapie der CIDP, hatten einen kontinuierlich fortschreitenden Krankheitsverlauf und hatten zuvor eine Behandlung mit Steroiden, IVIGs oder beiden Therapieoptionen erhalten, jedoch mit insuffizientem Ansprechen. Alle geeigneten Patienten, die ihr Einverständnis erteilten, wurden für die Studie ausgewählt. Die Daten der Studienteilnehmer wurden auf individuellen Datenerfassungsbögen erhoben. Ein Zyklus einer IA oder PLEX bestand aus 5 Behandlungen an 5 aufeinanderfolgenden Tagen. Als primärer Endpunkt wurde ein kombinierter CIDP Score verwendet, der aus 3 validierten Skalen bestand (Inflammatory Neuropathy Cause and Treatment (INCAT) Score, Motor Score (Medical Research Council, MRC) und Vibrationsempfinden (Stimmgabeltest)). Für kurzfristige Effekte wurden die absoluten CIDP Scores vor und nach jeder Behandlung verglichen, für langfristige Effekte die Veränderungen des CIDP-Scores pro Monat während IA oder PLEX im Vergleich zu vorangegangenen Therapien. Es erfolgte eine systematische Evaluierung von Nebenwirkungen (Adverse Events, AEs) sowie die Erhebung von Sicherheits-Labordaten und Immunglobulin-Reduktionsraten.

**Ergebnisse:** Insgesamt wurden 80 Patienten eingeschlossen, von denen 74 mindestens einen IA-Zyklus und 25 mindestens einen PLEX-Zyklus erhielten. 41 IA- und 16 PLEX-Patienten erhielten 2 oder mehr Zyklen (Median 4 (IQR 2-7.5), Maximum 43 Zyklen) über eine mediane Zeitspanne von 12.0 (6.0-34.0) Monaten in medianen Zeitabständen von 2.5 (1.9-4.3) Monaten. Wir beobachteten Verbesserungen des CIDP Scores nach den Behandlungen in der IA-Gruppe (mediane Verbesserung von 310 (224-374) auf 321 (234-373) Punkten; p<0.0001), aber nicht in der PLEX-Gruppe (Median 254 (214-358) vs. 254 (209-351), p=0.12). Langfristige Progressionsraten im Vergleich zu den vorhergehenden Kortikosteroid- und IVIG-Therapien sanken von 3.8 (2.2-9.1) auf 0.2 (-0.5-2.2) Punkte pro Monat in der IA-Gruppe (mediane Differenz 5.0, 95% KI 2.0-6.0, p<0.0001) und von 4.2 (2.6-17.2) auf -1.1 (-1.6-0.5) Punkte pro Monat in der PLEX-Gruppe (mediane Differenz 0.0, 95% KI 0.0-5.0, p=0.001), entsprechend einer klinischen Stabilisierung der Krankheitsprogression. Wir detektierten 12 (5.0%) asymptomatische und 3 (1.3%) symptomatische Jugularvenenthrombosen infolge der Anlage des Shaldon-Katheters in 240 IA-Zyklen sowie 6 Jugularvenenthrombosen (alle asymptomatisch) in 79 PLEX-Zyklen, was die Haupt-Komplikation beider Prozeduren repräsentierte. In beiden Gruppen waren Hypoproteinämie (IA 100%, PLEX 93.5%), Thrombozytopenie (IA 33.6%, PLEX 4.9%) und Anämie (IA 21.6%, PLEX 61.8%) die häufigsten Laborveränderungen während der Behandlung.

**Interpretation:** Die Ergebnisse zeigen, dass die wiederholte Anwendung von IA oder PLEX zu einer Stabilisierung der Erkrankung bei der Mehrzahl der Patienten führten und somit vielversprechende therapeutische Optionen bei Patienten mit CIDP mit unzureichendem Ansprechen auf Kortikosteroide und IVIGs darstellen könnten. Allerdings müssen die Invasivität des Verfahrens und das signifikante Risiko einer Jugularvenenthrombose berücksichtigt werden. Limitationen der Studie umfassen die nicht-randomisierte Gruppenzuteilung, die retrospektive Datenerhebung hinsichtlich der vorausgehenden Therapien, die Möglichkeit einer nicht optimalen Behandlung vor Baseline, die limitierte Patientenzahl und potentielle Störfaktoren (wie z.B. Cross-Overs zwischen IA und PLEX) durch die lange Observationszeit. Aufgrund dieser Limitationen erlauben die Studienergebnisse keinen direkten Vergleich der Wirksamkeit von IA und PLEX. Hierfür werden weitere randomisierte, kontrollierte Studien benötigt.

**Finanzierung:** Es handelt sich um eine investigator-initiated trial (IIT) ohne institutionelle oder industrielle Finanzierung.

**Schlüsselworte:** Chronische Inflammatorische Demyelinisierende Polyneuropathie, CIDP, Immunadsorption, Langzeiteffekte
